# Pre-processing Cooling of Harvested Grapes Induces Changes in Berry Composition and Metabolism, and Affects Quality and Aroma Traits of the Resulting Wine

**DOI:** 10.3389/fnut.2021.728510

**Published:** 2021-11-24

**Authors:** Margherita Modesti, Ron Shmuleviz, Monica Macaluso, Alessandro Bianchi, Francesca Venturi, Stefano Brizzolara, Angela Zinnai, Pietro Tonutti

**Affiliations:** ^1^Life Science Institute, Scuola Superiore Sant'Anna, Pisa, Italy; ^2^Department of Agricultural, Food and Agro-Environmental Sciences (DAFE), University of Pisa, Pisa, Italy; ^3^Interdepartmental Research Center, Nutraceuticals and Food for Health, University of Pisa, Pisa, Italy

**Keywords:** volatile organic compounds (VOCs), polyphenols, *Vitis vinifera*, Vermentino, postharvest, pre-cooling, sesquiterpenes

## Abstract

Due to the greenhouse gas increase, grapes are often exposed to high temperatures in several growing areas especially during the final developmental stages, and this is particularly true when early ripening cultivars are harvested. This may cause undesirable effects on berry metabolism and composition and wine quality, particularly concerning the aroma profile. Harvesting at night or keeping the harvested grapes in cold rooms before vinification are empirical protocols applied in specific viticultural areas. To study the effects of decreasing berry temperature after harvest, white-skinned berries (cv Vermentino) were maintained at 4 or 10°C for 24 or 48 h before processing (pre-cooling). Control grapes were kept at 22°C. Grapes cooled at 10°C for 24 and 48 h resulted richer in polyphenols and showed a significant up-regulation of genes involved in polyphenols biosynthesis (i.e., *VvPAL, VvSTS2*, and *VvFLS1)*. Similar behavior was observed in samples kept at 4°C for 48 h. Pre-cooling induced specific changes in the volatile organic compound (VOC) profiles. In particular, higher amounts of a specific subcategory of terpenes, namely sesquiterpenes, were detected in cooled samples. The induction of the expression of key genes involved in terpenoids biosynthesis (*VvHDR, VvDX3, VvTER, VvGT14*) was detected in cooled grapes, with variable effects depending on temperature and treatment duration. In both cooled samples, the evolution of alcoholic fermentation followed a regular trend but ended earlier. Higher phenolic content was detected in wines obtained from the 10°C-treated grapes. Higher residual concentration of malic acid at the end of fermentation was detected in wine samples from grapes pre-cooled at 4°C. Sesquiterpenes also showed a general increase in wines from cooled grapes, especially after pre-cooling at 10°C for 48 h. Different sensory profiles characterized the wine samples, with the best scores in terms of general pleasantness obtained by the wine produced from grapes pre-cooled at 4°C for 24 h. These results demonstrate that pre-cooling harvested grapes induces specific effect on the VOC profile and other quality parameters of Vermentino wine, and this appears to be the result of specific metabolic and compositional changes occurring in the berries.

## Introduction

The increase of average temperatures characterizing the last decades is affecting the grape-growing and wine sectors (1). High temperatures at or after veraison, accompanied by a limited thermal fluctuation between day and night, induce metabolic unbalances such as the acceleration of sugar and organic acid metabolism, altered tannin and anthocyanin synthesis, and the desynchronization of technological and phenolic maturity (2, 3). Moreover, high temperatures occurring at ripening and in correspondence of harvesting are recognized to decrease the aromatic potential of the berries by affecting aroma and aroma precursor-related biosynthetic genes (e.g., terpenoids) (3) and inducing oxidative losses (4). Additionally, crushing grapes at too high temperatures potentially induces spontaneous fermentations. To limit these problems, harvest, in some viticultural areas, is performed at night or harvested grapes are kept overnight or up to 24–48 h in cold rooms at temperatures ranging between 4 and 10°C before crushing (pre-processing cooling). The main goal of this technical approach is that of removing heat from the berries. The effects of these protocols are empirically reported by several winemakers in terms of a better control over the fermentation process, an increased complexity of aromatics, and a vibrant acidity of the resulting wines. There is a vast technical and scientific literature concerning the refrigeration of harvested fruits as an approach resulting in the delay of ripening and senescence, thus prolonging commercial/shelf life of perishable products, including table grapes (5). Considering pre-processing cooling of wine grapes, little information is available, and the (few) published papers mainly report the effects on the wines. Heredia et al. (6) indicated that Syrah red wines show intense and stable color traits after keeping grapes in a cold-storage room (below 4°C) for 24 h prior to crushing and successive cold maceration at 3–8°C. Although effects of pre-processing cooling may vary in relation to the field or pre-harvest conditions and the vinification protocols (e.g., yeast strains used), the reduction of the grape temperature appeared to positively affect Sauvignon blanc wine quality parameters (7, 8) with significant impact in terms of aroma classes (esters, monoterpenes) depending on the pre-crushing temperatures of the grapes. The same author showed that keeping grapes (cv. Pinotage) overnight at 10°C and then maintaining the same temperature during skin contact with the must prior to fermentation resulted in the production of the most typical and highest quality Pinotage wines (9). Mafata et al. (10) reported that the sparkling wine produced with Chardonnay and Pinot Noir grapes stored at 0 and 10°C had more desirable aroma attributes, particularly fruity, fresh, and floral attributes, compared to wines from higher temperature treatments. These effects might be the result of either changes in the metabolism and composition of the berries during the cooling treatment, or adjustments of the crushing and fermentation processes. Considering the effects on grapes, Coletta et al. (11) reported that, in skin of cv Falanghina berries, the activity of cell wall pectin-methylesterase is differently affected by thermal pre-processing treatments of the grapes. In a recent report, Modesti et al. (12) showed that cv Vermentino berries react to a short-term refrigeration (4 or 10°C) pre-crushing treatment by increasing the concentration of terpenoids, but no information has been provided concerning the resulting wines. These data indicate that grape berries specifically react to the imposed stress condition by modifying specific physiological and metabolic processes, resulting in compositional changes, and impacting the quality of the wines. In this context, based on the hypothesis that compositional modifications induced by pre-processing refrigeration could potentially improve grapes and wine quality, our main goal was to evaluate the effect of short-term low temperature conditioning on volatile organic compound (VOC) profile and wine sensory traits. Therefore, here, we report both the effects of applying pre-processing cooling (pre-cooling) on specific molecular and metabolic parameters of grapes (cv Vermentino) and the quality traits of the resulting wines.

## Materials and Methods

### Grape Samples and Low Temperature Treatments

Bunches of white-skinned grapes (*Vitis vinifera* L.), cv Vermentino, were hand harvested, collecting bunches from nine grapevines (from three adjacent panels) at an average total soluble solids (TSS) value of 22 (±0.1) °Brix. The commercial vineyard (Lodolina) is located in Candia hills (Massa province, Tuscany, Italy. 44°02′197.6″ N, 10°11′265.9″ E) in calcareous soils and with favorable exposure (north–south aligned rows). Vines are trained at simple Guyot, and all agronomic practices follow the disciplinary of production for the Appellation of Controlled Origin (DOC) Candia dei Colli Apuani. After harvest, grapes were selected based on homogeneous bunch shape and the absence of visual defects or evident diseases, and immediately transported to the laboratory. Grapes were divided into six different lots (5 kg each) and were placed in perforated plastic cassettes (60 × 40 × 14 cm) in a single layer. Two lots were kept in an airconditioned room at 22°C (±0.5) for 24 (22°C 24) and 48 (22°C 48) h and used as control. The other lots were kept in cold rooms under different temperature conditions: two lots were refrigerated at 4°C (±0.5) for 24 (4°C 24) and 48 (4°C 48) h, and another two lots were refrigerated at 10°C (±0.5) for 24 (10°C 24) and 48 (10°C 48) h. Berries were sampled in triplicates at harvest (T0) and after the treatments for the specific analyses. Grapes from T0 and from all the cooling treatments (4 and 10°C) were processed to make wine.

### Laboratory Scale Fermentation

**Figure 1:**
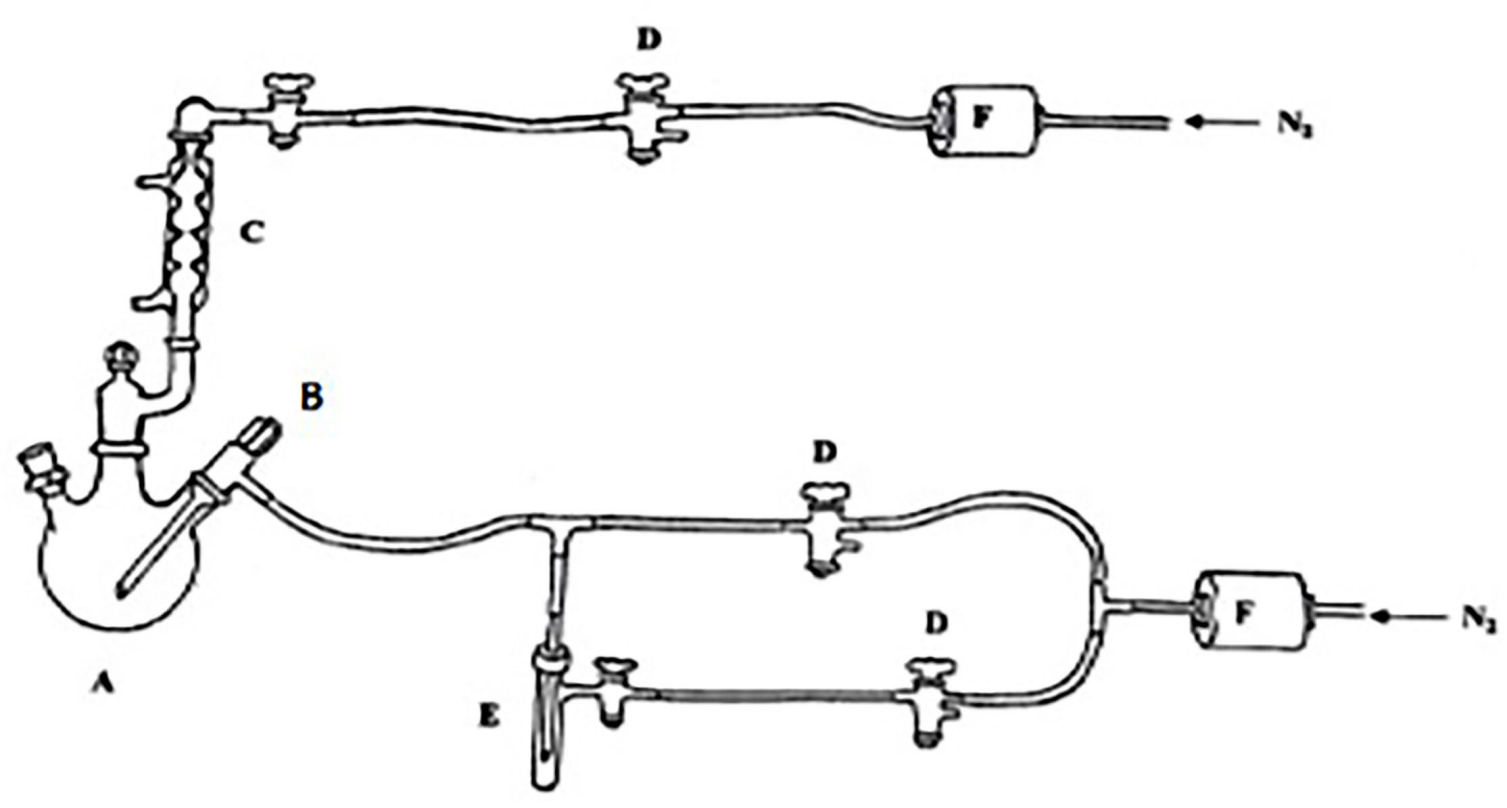
Scheme of the wine production technical setup: **(A)** bio fermenter; **(B)** float for sampling; **(C)** coolant; **(D)** three-way taps; **(E)** sample collection tube, and **(F)** sterilizing gas filters.

Micro vinifications were carried out in a lab-scale plant, following standard procedures for white wine. A purpose-built experimental apparatus was used in order to protect the environment from any external contamination. As illustrated in Figure 1, the system consists of a 4 L Pyrex glass flask equipped with three necks, with a bubble cooler, a thermometer, and a dip tube connected. This system is used for taking an aliquot of the sample. The presence of an overpressure of nitrogen inside the equipment with a filter (0.2 μm) prevents the access of possible external contaminants. The different components of the bioreactor were assembled after sterilization with a Bunsen burner and under a sterile N_2_ flow. To ensure temperature control during the fermentation process, the flask was immersed in a chilled liquid by a control unit (20°C). The must and the yeasts (6.5 g/kg of UVAFERM 43-LALLEMAND) were introduced inside the bio fermenter at room temperature and pressure.

### Grapes Technological Parameters

For technological parameters analyses, 30 berries were randomly sampled in triplicate after the harvest (T0) and at the end of each treatment. The collected berries were immediately pressed and the obtained must was centrifuged (8,000 rpm for 5 min at 22°C) and filtered with syringe filters (0.22 μm pore size, 33 mm diameter, SigmaAldrich, Italy). The centrifuged and filtered must was then used for pH measurement using a pH meter (pHmetro GLP21; Crison Instruments), TSS using an optical refractometer (the obtained °Brix transformed in g/L of hexose), and titratable acidity (TA) by titrating 7.5 ml of must with 0.1 N sodium hydroxide (NaOH) and expressed in g/L of tartaric acid. For each treatment, 30 additional berries were collected and powdered with liquid nitrogen. A total of 250 mg of powdered tissue was used to extract total polyphenols, adding 1.25 ml of methanol (80%), and centrifuged at 4°C, 10,000 rpm for 15 min. The Folin Ciocalteau method was used to measure total polyphenols content (TPC) (13) by adding 300 μl of sodium carbonate at 7.5%, 100 μl of Folin–Ciocalteau reagent (Sigma-Aldrich, Italy) to 20 μl of supernatant, and expressed as mg of gallic acid equivalents (GAE) × 100 g^−1^ fresh weight. Lastly, bunches from each treatment were tagged and weighed at T0 and throughout sampling to monitor the weight loss (WL).

### Wine Chemicals Analysis

The sugar, organic acid, and ethanol content of musts and wines were determined using specific commercial enzymatic kits (14). Total titratable acidity, pH, volatile acidity, and total phenols were measured as previously reported (15).

### Determination of Wine Color Coordinates

For the determination of the chromatic characteristics, a Benchtop CLM-196 colorimeter [Eoptis-38121 Trento (TN)-ITALY] was used. The instrument interfaces through the USB port to a PC with Microsoft Windows operating system. The acquired color values are expressed using the native CIE coordinates L ^*^ a ^*^ b according to the official OIV-method International Organization of Vine and Wine (OIV) (16). The characterization of color in the CIELAB space can also be done using the so-called cylindrical coordinates, h ^*^ and C ^*^, also used in the present trial. The first defines the psychometric hue while the second defines the psychometric chroma which are related to the perceptual term hue and saturation, respectively.

### Sensory Analysis

The sensorial profiles of wine, as a function of the glass utilized for tasting, were carried out by a trained panel (eight assessors, five males and three males aged between 28 and 65 years). All the assessors were included in the “expert panel” of the Department of Agriculture, Food and Environment (DAFE) of the University of Pisa, and had a previous experience in sensory descriptive analysis, mainly in wine evaluation. According to the DAFE internal procedure for assessor selection and training (17), expert assessors must repeat and pass re-qualification tests at least once a year to demonstrate that they are still capable of evaluating the samples satisfactorily.

The assessors used a sensorial sheet specifically developed for this purpose consisting of a not structured, parametric, descriptive wine scoring chart. Supplementary Table 1 reports the synthetic definition of each descriptor present in the sensorial sheet shown to the assessors before starting the sensorial evaluations, with the aim to clearly define the meaning of the terms proposed.

The panelists commented the qualities and evaluated the intensity of each parameter on a continuous scale of 0–10, including visual, aroma, taste attributes, and a hedonic parameter such as the overall appreciation.

Tasting was carried out in the morning, in a well-ventilated quiet room and in a relaxed atmosphere.

Each sample was labeled with a three-digit code randomly assigned and was served to all the assessors by a trained sommelier that was not included in the panel involved in experimental tasting sessions.

The wine samples were assessed by the panelists at the same time in the different glasses.

### Gene Expression Analyses

At T0 (harvest) and at the end of each treatment, thirty berries were collected in triplicate and the skin was separated from the pulp and immediately frozen in liquid nitrogen. Frozen skins were grounded to powder using ceramic mortar and pestle pre-cooled with liquid nitrogen. One hundred milligrams of grounded tissue was used for total RNA extraction using Spectrum™ Plant Total RNA Kit (Sigma-Aldrich, Italy), including DNA digestion with the On-Column DNase I Digestion Set (Sigma-Aldrich, Italy). RNA concentration and purity were determined with Nanodrop 2000 spectrophotometer (Thermo Scientific, Italy), verifying an absorbance ratio 260/280 nm between 1.8 and 2 and 260/230 nm between 1.3 and 2. Integrity of the RNA extracted was checked on a 1 % (weight/volume) agarose gel. Reverse transcription of the RNA templates to cDNA was carried out using 50 ng of RNA and 4 μl of ReadyScript™ cDNA Synthesis Mix (Sigma-Aldrich, Italy). Double-distilled water (DDW) (Sigma-Aldrich, Italy) was used to reach a total volume of 20 μl. The PCR conditions were set according to the manufacturer protocol. *Alpha-terpineol synthase* (*VvTER*), *phenylalanine ammonia lyase* (*VvPAL*), *flavonol synthase 1* (*VvFLS1*), *stilbene synthase 2* (*VvSTS2*), *polyphenol oxidase* (*VvPPO*), *deoxy-D-xylulose 5-phosphate synthase 3* (*VvDXS3), 4-hydroxy-3-methylbut-2-enyl diphosphate reductase* (*VvHDR)*, and *monoterpene beta-D-glucotransferase 14 (VvGT14*) were selected as marker genes for molecular investigations of specific metabolic pathways after the treatments. The sense and antisense primers were designed with the primer designing tool of NCBI, based on the mRNA sequences of the target genes from the *Vitis vinifera* genome present in GenBank. The primer couples were inserted into the NCBI Basic Local Alignment Search Tool (BLAST) in order to verify a specific amplification. Primers were synthesized by Sigma-Aldrich (Italy). Before sample analyses, the amplification efficiency of each couple of primers was determined with a standard curve generated using a serial dilution of representative cDNA mixture. The cDNA template was diluted three-fold for less expressed genes and six-fold dilution was applied for more expressed genes. A range of acceptable efficiencies have been determined (90–110%) for further analysis (18). The forward and reverse sequences, GenBank Accession, reaction temperature, and primer efficiencies are given in Supplementary Table 2. For samples analyses, RT-qPCR was performed using the SYBR Green PCR Master Mix (Life Technologies™) with a final reaction volume of 10 μl running on the CFX Connect Real-Time PCR System (BioRad^©^). The RT-qPCR cycle was set as follows: initial denaturation at 95°C for 2 min, followed by 40 cycles of amplification with denaturation at 95°C for 15 s and annealing and elongation at specific temperatures suited for each couple of primers for 1 min. Following the 40 cycles, a melt cycle was performed at 95°C for 15 s and 60°C for 15 s to detect possible primer dimers or nonspecific amplification in cDNA samples. PCR reactions were run in triplicate and a negative control of the PCR mix in addition to the primers was performed in all qPCR runs. Results were processed with the comparative Ct method (18) employing *Actin 7* (*VvACT7*) as housekeeping gene. The relative quantification of each gene tested was calculated using the 2^−ΔΔCt^ method.

### HS-SPME GC-MS Analysis

For VOCs analysis, 30 berries per biological replicate (five biological replicates for each treatment) were homogenized and a NaCl buffer solution (1 M) has been added (1:1 w/w) by using an UltraTurrax (Mod. T25, IKA), immediately frozen in liquid nitrogen, and stored at −80°C for further analysis. The pre-homogenized berries were thawed at 15°C for 15 min and 10 g were weighed in a 20 ml glass crimp vial for headspace analysis (Cat. No. SU860049, SigmaAldrich, Italy) sealed with silicone septa for SPME (Cat. No. 27362, SigmaAldrich, Italy). For wine analyses, 6 gr of wine were mixed with 1 g of NaCl in a 20 ml glass crimp vial for headspace analysis and sealed with silicone septa for SPME. Five technical replicates were run for each treatment. Grapes samples were incubated under agitation for 45 min at 40°C. Wine samples were incubated under agitation for 30 min at 40°C. VOCs were sampled at the same temperature for 45 and 30 additional min, respectively for grapes and wine, using an SPME fiber (50/30 μm, DVB/CAR/PDMS, 1 cm; Supelco, Bellefonte, PA, USA). The fiber was then desorbed for 5 min into the injector of the GC set at 250°C. A Clarus 680 Gas Chromatograph equipped with a split/splitless injector (PerkinElmer^®^, Waltham, Massachusetts) was employed for the analysis. Volatiles were separated on a fused silica capillary column (DB Wax, 60 m, 0.32 mm ID, 0.25 μm film thickness. Restek, Bellefonte, PA). Helium with a flow rate of 1 ml min^−1^ was used as carrier gas. The GC-MS settings employed were the same adopted by Genova and Montanaro (19). For compounds identification, mass spectrometer (Clarus 500 Mass spectrometer, PerkinElmer^®^, Waltham, Massachusetts) was used. Each chromatogram was run on AMDIS software (National Institute of Standards, Gaithersburg, MD, USA) and each peak was identified by comparing the spectra with those of the National Institute for Standards and Technology (NIST 98, Version 2.0, USA) data bank. Only compounds with 75% of identity or more were selected. Selected peaks were then quantified using TurboMass software (TurboMass^®^, Version 5.4.2 PerkinElmer Inc., USA, 2008), by integration of the areas of the peak. The area of each peak was normalized on the sum of the areas to eliminate variations due fiber decay.

### Statistical Analyses

Each set of replicates was tested to detect outliers through a principal component analysis (PCA). All data were then statistically analyzed through Shapiro-Wilk and Bartlett test to verify normality and homogeneity of variances. Once these pre-requisites were established, collected data were compared separately for each sampling time (24 and 48 h) by one-way ANOVA test and Tukey's honestly significant difference (HSD) test with *p* ≤ 0.05 for multiple comparison. Additionally, one-way ANOVA was run to compare data collected at T0 and after the different treatments. The statistical tests were performed using GraphPad Prism version 7.0 (GraphPad Software, La Jolla California USA). VOCs levels were then transformed as fold change values by normalization to their level at harvest time (T0) and transformation in logarithmic scale as following: log_2_ (FC) = log_2_ [replicate/mean (T0)]. A hierarchical clustering analysis (HCA) was performed on the log_2_ (FC) of the features that were significantly different comparing treated grapes and T0 samples employing a Pearson correlation coefficient using average as clustering algorithm. The results were used to create heatmaps. Regarding wine, all the detected features have been included in the reported HCA. All multivariate analyses were performed employing R Studio (Version 1.4.1106,^©^ 2009–2021 RStudio PBC).

## Results

### Grapes Technological Parameters

All samples lost weight after 24 and 48 h (Table 1). As expected, the percentage of weight loss was higher in grapes kept at 22°C (control grapes) compared with grapes from both cooling treatments, with statistically significant difference observed after 48 h. Compared to grapes at harvest (T0), the pH values were not statistically different. All the samples showed, compared to T0, a general decrease of titratable acidity, with the only exception represented by the grapes kept at 4°C for 24 h, which showed a TA level similar to that recorded in grapes at harvest. In cooled grapes, TA values resulted, as a trend, higher than those observed in the respective control samples. As expected, due to the concentration effect (higher water loss), TSS content showed the highest values in both control samples, while the lowest values were detected in grapes refrigerated at 4°C for 24 h and at 10°C for 48 h. Compared to T0, TPC significantly increased in grapes cooled for 48 h at 10°C, and a similar trend was observed in grapes cooled at the same temperature for 24 h, while the lowest values were observed in control berries at both sampling time (24 and 48 h).

**Table 1:** Technological parameters in grapes (*Vitis vinifera L*. cv Vermentino) at harvest (T0) and after the following postharvest cooling treatments: 4 and 10°C for 24 h (4°C 24 and 10°C 24) and 48 h (4°C 48 and 10°C 48).

	**T0**	**4^**°**^C 24**	**10^**°**^C 24**	**22^**°**^C 24**	**4^**°**^C 48**	**10^**°**^C 48**	**22^**°**^C 48**
Weight loss (%)	–	0.90 ± 0.40 c	0.91 ± 0.28 c	2.04 ± 0.74 bc	2.84 ± 0.95 b	2.22 ± 0.27 b	5.00 ± 1.50 a
pH	3.42 ± 0.02 ab	3.38 ± 0.02 b	3.43 ± 0.01 a	3.46 ± 0.01 a	3.42 ± 0.01 a	3.36 ± 0.02 b	3.49 ± 0.01 a
TA (g/L Tartaric acid)	5.39 ± 0.03 a	5.52 ± 0.03 a	4.85 ± 0.05 bc	4.56 ± 0.07 c	4.55 ± 0.02 c	4.96 ± 0.05 b	4.46 ± 0.04 bc
TSS (g/L Hexose)	217.2 ± 1 b	194.2 ± 1 d	214.8 ± 4 b	221.7 ± 3 a	202.2 ± 1 c	193.1 ± 1 d	226.4 ± 1 a
TPC (GAE mg/L)	758 ± 46 bcd	668 ± 63 cde	792 ± 57 ab	596± 55 e	697 ± 59 bcde	880 ± 74 a	609 ± 46 e

*22°C refers to control samples after 24 and 48 h (22°C 24 and 22°C 24). Different letters indicate statistically significant differences at p ≤ 0.05 according to the results of one-way ANOVA and Tukey's HSD test. Values are the mean of three biological replicates ±SD*.

### Grape VOC Profile

**Figure 2:**
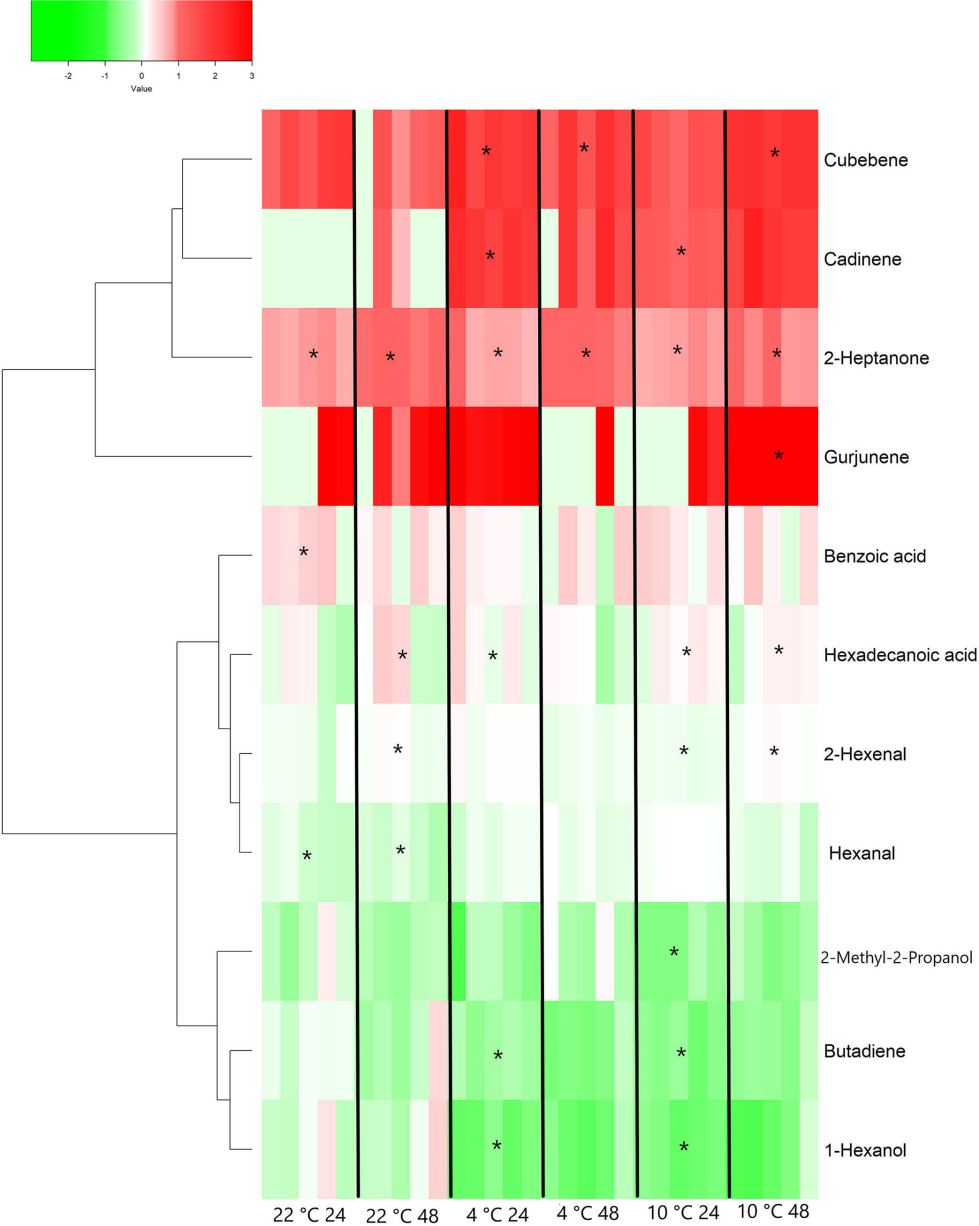
Heatmap and Hierarchical Cluster Analysis performed on volatile organic compounds (VOCs) measured in Vermentino grapes following cooling treatment at 4 and 10°C for 24 and 48 h and in control grapes at 22°C at the same sampling times. The heatmap shows the fold change of identified VOCs. Each cell represents the level of each compound in each of the five biological replicates for the specific postharvest condition, normalized to its level at harvest (T0) and transformed in logarithmic scale as following: log_2_(FC) = log_2_[replicate/mean(T0)]. A Pearson correlation coefficient was employed in order to group the different features, using average as clustering algorithm. Asterisks represent statistically significant differences compared to T0 samples (based on ANOVA and Tukey *post-hoc* test with *p* ≤ 0.05 performed separately for each sampling times).

The HS-SPME GC-MS analysis identified a total of 17 VOCs in the sampled grape berries. Among them, 10 compounds (cadinene, hexadecenoic acid, butadiene, 2-heptanone, 1-hexanol, hexanal, cubebene, 2-hexenal, 2-methyl-2-propanol and benzoic acid) resulted significantly different when comparing treatments after 24 h. Cadinene, hexadecanoic acid, hexenal, butadiene, and 1-hexanol resulted to be also significantly different for both low temperatures from the respective control at 22°C for 24 h. On the other hand, after 48 h of treatments, just five compounds (2-heptanone, hexadecanoic acid, gurjunene, cubebene and 2-hexenal) resulted statistically different among the samples and/or from the T0 grapes. Cubebene was the only compound significantly higher in both cooled samples (48 h) than T0 and the respective control at 22°C (statistical analysis provided in Supplementary Tables 3 and 4 for 24 and 48 h samples, respectively). Considering 24 and 48 h data together, the VOCs identified at both sampling times and as statistically different comparing with T0 grapes were transformed as fold change values by normalization to their level in samples at harvest time (T0). An HCA was performed with the log_2_ (FC) data in order to group the different features. The results are presented in the heatmap reported in Figure 2. The clustering algorithm grouped together 3 terpenes (cadinene, cubebene and gurjunene) and 2-heptanone, all showing an increasing trend compared to T0. Cubebene and cadinene significantly increased compared to T0 and to control in 48 and 24 h samples, respectively. Gurjunene resulted to be higher than T0 and control only in 10°C 48 samples. 2-heptanone showed increased levels compared to T0 also in control grapes, suggesting that this behavior was temperature-independent and potentially related to a time effect. Control grapes also showed a significant accumulation of benzoic acid and a reduction of hexanal after 24 h. In these latter samples, an increase of hexadecanoic acid and 2-hexanal has also been observed after 48 h. A similar trend was detected in 4°C in 24 samples and in grapes kept at 10°C for 24 or 48 h. An additional cluster is formed by 2-methyl-2-propanol, butadiene, and 1-hexanol that show a general decreasing trend compared to T0 sample. Butadiene and 1-hexanol resulted to be significantly lower than T0 and control in both cooled samples for 24 h (Figure 2).

### Relative Gene Expression Level

**Figure 3:**
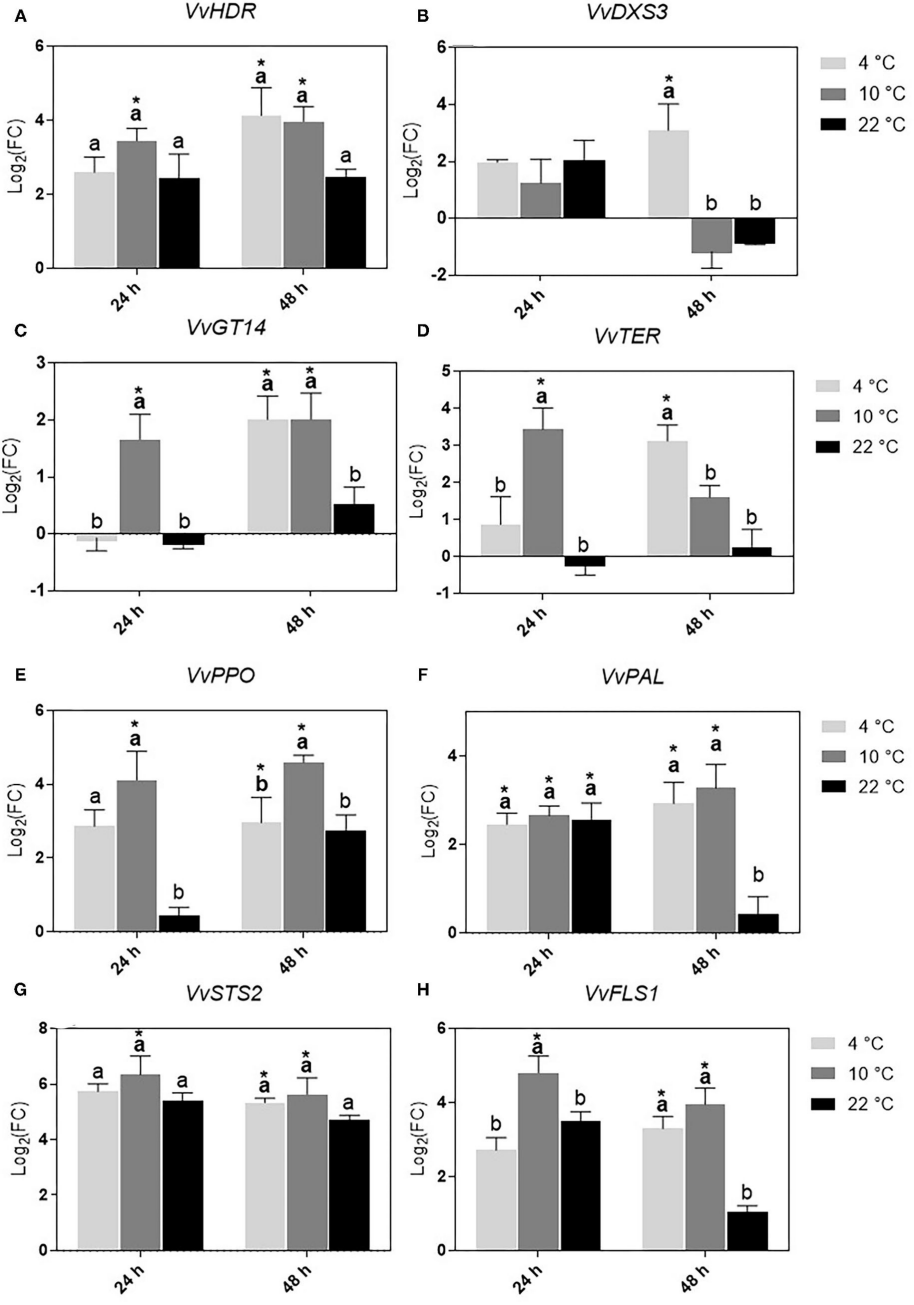
Relative expression of *VvHDR* **(A)**, *VvDXS3* **(B)**, *VvGT14* **(C)**, and *VvTER* **(D)**, *VvPPO* **(E)**, *VvPAL* **(F)**, *VvSTS2* **(G)**, and *VvFLS1* **(H)** genes over sampling time in Vermentino berry skins following cooling treatment of grapes at 4 and 10°C for 24 and 48 h and in control grapes at 22°C at the same time periods. The gray color scale represents different treatment temperatures (4, 10, and 22°C). Each bar represents the average of the expression level in the three analyzed biological and two technical replicates, normalized to the expression level at harvest (T0) and transformed in logarithmic scale as following: log_2_(FC) = log_2_[replicate /mean(T0)]. Different letters indicate statistically significant differences (*p* ≤ 0.05) according to the results of the Tukey's HSD test performed between treatments separately in each sampling time. Asterisks indicate statistically significant differences (*p* ≤ 0.05) according to the results of the Tukey's HSD test performed between treatments and T0 grapes.

The results for the relative expression of key genes involved in volatile terpenoids biosynthetic pathways, and polyphenols biosynthesis and metabolism in the skin are presented in Figure 3. The reported expression level is normalized to the expression level at harvest (T0) and transformed in logarithmic scale. Considering specific terpene biosynthesis-related genes, *VvHDR* was significantly up-regulated in cooled grapes at 10°C after both 24 and 48 h of treatment compared to T0 (Figure 3A). A significant increase of the expression of this gene was also detected in cooled samples at 4°C for 48 h. This sample was the only one showing an up-regulation of *VvDXS3* (Figure 3B). *VvGT14* (Figure 3C) and *VvTER* (Figure 3D) were up-regulated in 10°C in 24 grapes and 4°C in 48 grapes. The former gene was significantly up-regulated in cooled grapes at 10°C for 48 h as well. Considering genes involved in polyphenol metabolism, following the treatment at 10°C for 24 and 48 h, *VvPPO* (Figure 3E) was significantly up-regulated compared to both T0 and control grapes. Cooled grapes at 4°C for 24 h showed an increase of the *VvPPO* expression level, but the difference was statistically significant only in comparison with control samples and not with T0. *VvPAL* (Figure 3F) showed an overexpression in all samples with the exception of control grapes at 48 h. The expression of *VvSTS*2 (Figure 3G) generally increased in all the applied conditions, but only in grapes refrigerated at 10°C for 24 and 48 h and at 4°C for 48 h was significantly different compared to T0 samples. The expression of *VvFLS1* (Figure 3H) followed a similar trend, with both samples cooled at 10°C and grapes kept at 4°C for 48 h showing a significant up-regulation.

### Wine Chemical Parameters

**Figure 4:**
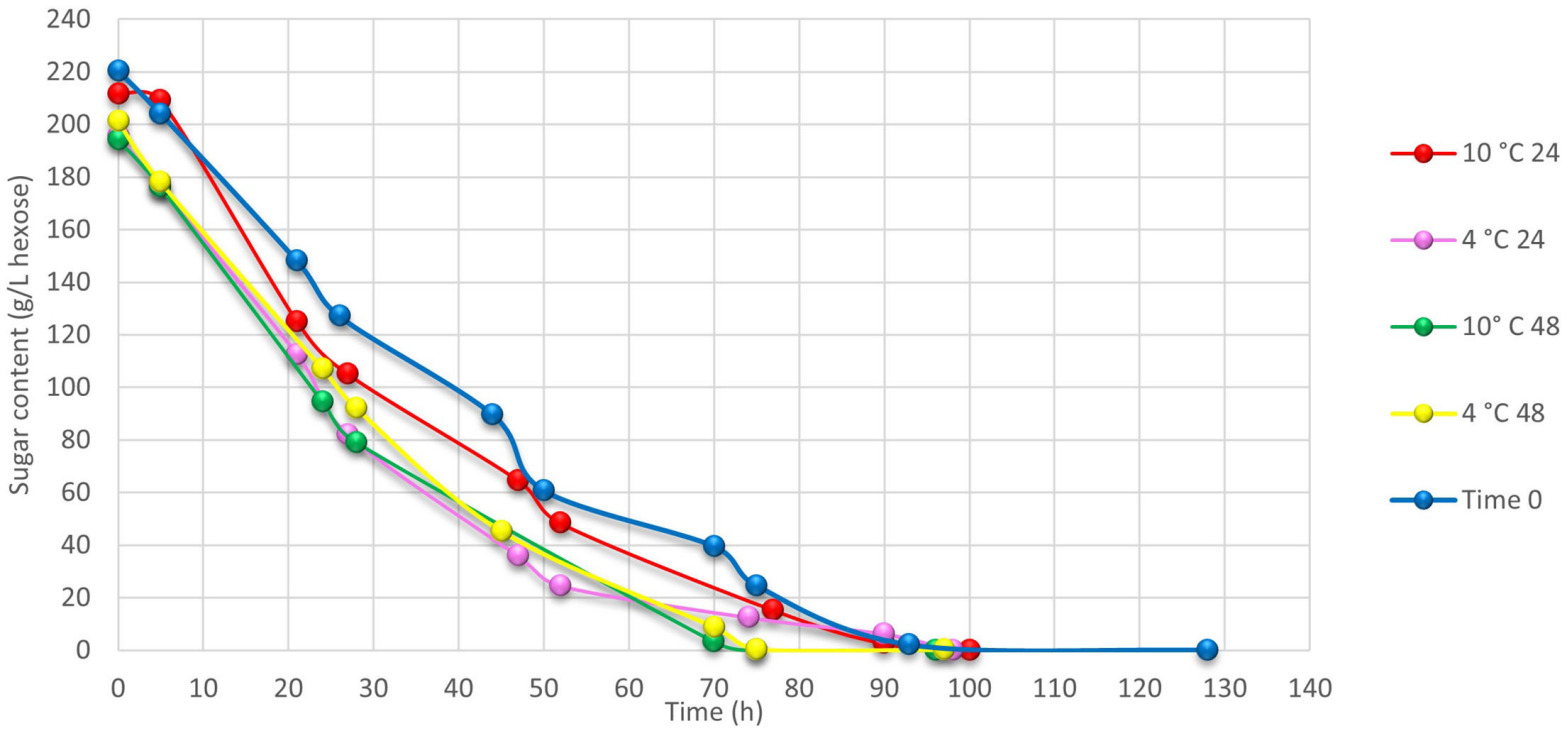
Changes of sugar content during alcoholic fermentation process in grape samples kept at 4 or 10°C per 24 and 48 h and at harvest (T0).

The alcoholic fermentation was monitored by determining the sugar content trend (g/L hexoses). All samples reached a final sugar content of <0.1 g/L regardless the cooling treatments and, therefore, all of them can be considered dry wines according to current OIV regulation (20). Compared to wine obtained from T0 grapes, in all cooled samples, the alcoholic fermentation followed a regular progress and finished earlier (Figure 4). At the end of the alcoholic fermentation every wine showed a chemical profile specific as a function of the treatment carried out. Among all the technological parameters investigated, some of them can better describe the effects of the pre-cooling treatments. As shown in Table 2, the pH slightly but significantly decreased in all cooled samples if compared with T0. The volatile acidity, related to the acetic acid content, was significantly lower in the sample refrigerated at 4°C for 24 h. Increasing trends of this parameter were observed in both samples kept at 10°C. The lower the temperature applied, the higher the residual concentration of malic acid at the end of alcoholic fermentation. Accordingly, T0 sample showed a lower acidic profile for a white wine, reaching the lowest titratable acidity level. All the samples treated with low temperature have developed a lower alcohol degree than the wine made from T0 grapes, consistently with the starting sugar content (Table 1). At the end of the alcoholic fermentation, total polyphenols content resulted higher in wines made from refrigerated grapes at 10°C (Table 2) which is consistent with the higher level of total polyphenols observed in these grapes (Table 1). Furthermore, wine made starting from refrigerated grapes at 4°C for both 24 and 48 h resulted richer of total polyphenols comparing with T0 wines. The technological parameters of the musts before the start of fermentation are shown in Supplementary Table 5.

**Table 2:** pH, titratable acidity (g/L tartaric acid), malic acid (g/L), alcohol degree (% V/V), volatile acidity (g/L), and polyphenols content (mg/L GAE) in wines at the end of the alcoholic fermentation.

	**T0**	**4^**°**^C 24**	**10^**°**^C 24**	**4^**°**^C 48**	**10^**°**^C 48**
pH	3.46 ± 0.02 a	3.40 ± 0.03 b	3.42 ± 0.02 b	3.43 ± 0.02 b	3.41 ± 0.03 b
TA (g/l tartaric acid)	5.55 ± 0.04 d	6.34 ± 0.06 a	6.10 ± 0.04 bc	6.04 ± 0.05 c	6.16 ± 0.08 b
Malic acid (g/L)	1.47 ± 0.05 c	1.90 ± 0.03 a	1.67 ± 0.05 b	1.95 ± 0.06 a	1.62 ± 0.04 b
Alcohol degree (% V/V)	13.0 ± 0.1 a	11.5 ± 0.1 d	12.5 ± 0.1 b	11.8 ± 0.1 c	11.4 ± 0.1 d
Volatile acidity (g/L)	0.26 ± 0.02 ab	0.16 ± 0.03 c	0.31 ± 0.02 a	0.20 ± 0.03 b	0.35 ± 0.03 a
Total polyphenols content (mg/L GAE)	573 ± 33 c	674 ± 37 b	801 ± 23 a	683 ± 34 b	832 ± 27 a

*Wines were made from Vermentino grapes immediately after harvest (T0) and following cooling treatment at 4 and 10°C for 24 and 48 h. Different letters indicate statistically significant differences at p ≤ 0.05 according to the results of one-way ANOVA and Tukey's HSD test. Values are the mean of three technical replicates ±SD*.

### Wine Color

The evolution of the CIELAB color scale was evaluated on musts and wines. As shown in Supplementary Table 6, all the musts are located in the area of 100° of hue angle (h^*^) that belongs to the medium yellow or with a very slight tendency to the green. Also, this grouping is given in an area that is very close to the origin of coordinates, with low values of chroma C^*^, that is with a high proportion of white light transmitted by the samples. These data, besides the levels of lightness (L^*^) near to 80% of transmitted global light quantity, determine the final color in the category of pale yellow. The values at end of fermentation (Table 3) show, in wines from cooled grapes, increases of the chroma C^*^ and decreases of the hue (h^*^) values, thus determining the final color in the category of the pale yellow with golden reflections, particularly in both 10°C samples.

**Table 3:** Wine CIELAB parameters (L*, a*, b*, C*, h*) at the end of the alcoholic fermentation.

	**T0**	**4^**°**^C 24**	**10^**°**^C 24**	**4^**°**^C 48**	**10^**°**^C 48**
L*	84.73 ± 0.12 a	84.56 ± 0.17 a	84.77 ± 0.13 a	84.85 ± 0.15 a	84.52 ± 0.16 a
a*	−0.42 ± 0.02 c	−0.03 ± 0.01 b	0.02 ± 0.01 a	−0.06 ± 0.02 b	0.01 ± 0.01 a
b*	3.53 ± 0.04 c	4.02 ± 0.03 b	4.20 ± 0.04 a	4.09 ± 0.04 b	4.28 ± 0.03 a
C*	3.55 ± 0.06 c	4.02 ± 0.04 b	4.20 ± 0.05 a	4.09 ± 0.06 b	4.28 ± 0.04 a
h*	96.8 ± 0.1 a	90.4 ± 0.3 b	89.7 ± 0.2 c	90.8 ± 0.2 b	89.9 ± 0.1 c

*Wines were made from Vermentino grapes at the harvest (T0) and following cooling treatment at 4 and 10°C for 24 and 48 h. Different letters indicate statistically significant differences at p ≤ 0.05 according to the results of one-way ANOVA and Tukey's HSD test. Values are the mean of three technical replicates ±SD*.

### Wine VOC Profile

**Figure 5:**
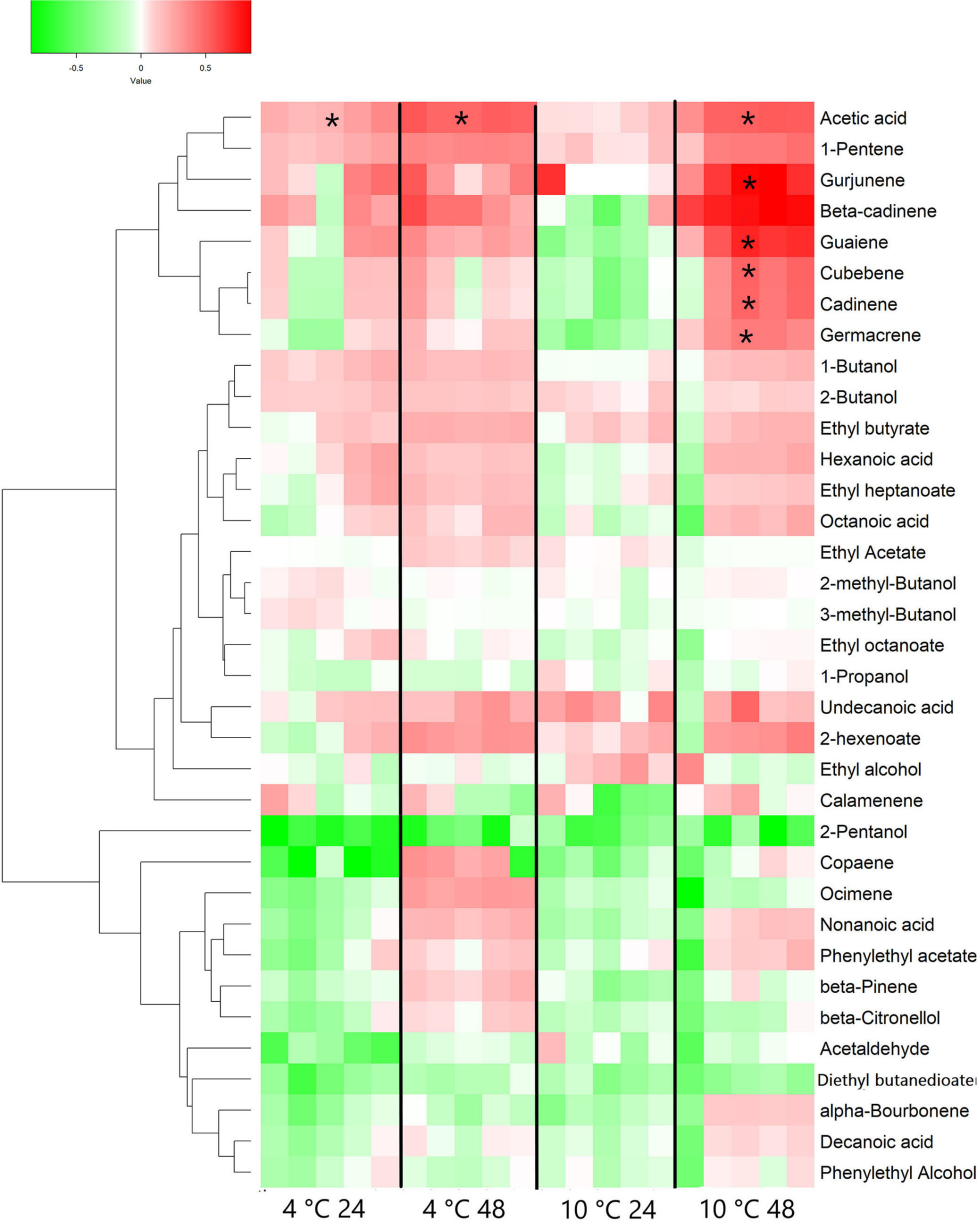
Heatmap and Hierarchical Cluster Analysis performed on VOCs measured in wine made from Vermentino grapes following cooling treatment at 4 and 10°C for 24 and 48 h. The heatmap shows the fold change of identified VOCs. Each cell represents the level of each compound in each of the five analyzed replicates for the specific postharvest condition, normalized to its level at harvest (T0) and transformed in logarithmic scale as following: log_2_(FC) = log_2_[replicate/mean(T0)]. A Pearson correlation coefficient was employed in order to group the different features, using average as clustering algorithm. * Represents statistically significant difference based on ANOVA and Tukey *post-hoc* test with *p* ≤ 0.05 performed between treatments and T0 grapes.

A total of 35 volatile compounds were identified in wine samples. A specific analysis has been performed on the dataset containing the volatiles identified in wines from both sampling times and temperatures. VOCs identified were transformed as fold change values by normalization to their level at harvest time (T0) and the results are presented as heatmap (Figure 5). A group of eight compounds (top of the heatmap) clustered together, showed a general increase marked in 4 and, particularly, 10°C 48 samples. Based on the univariate statistical analysis performed separately for each sampling time, among these compounds, only acetic acid resulted statistically different between the 4°C in 24 and 48 samples and T0 grapes, while acetic acid, again, and the sesquiterpenoids gurjunene, guaiene, cubebene, cadinene, and germacrene were statistically different between T0 and the 10°C in 48 samples only (ANOVA details in Supplementary Tables 7, 8). A group of clustered compounds (bottom of the heatmap) include metabolites (some of them belonging to monoterpene class) showing, in general, a decreasing trend in wine samples obtained from refrigerated grapes.

### Sensory Analysis

**Figure 6:**
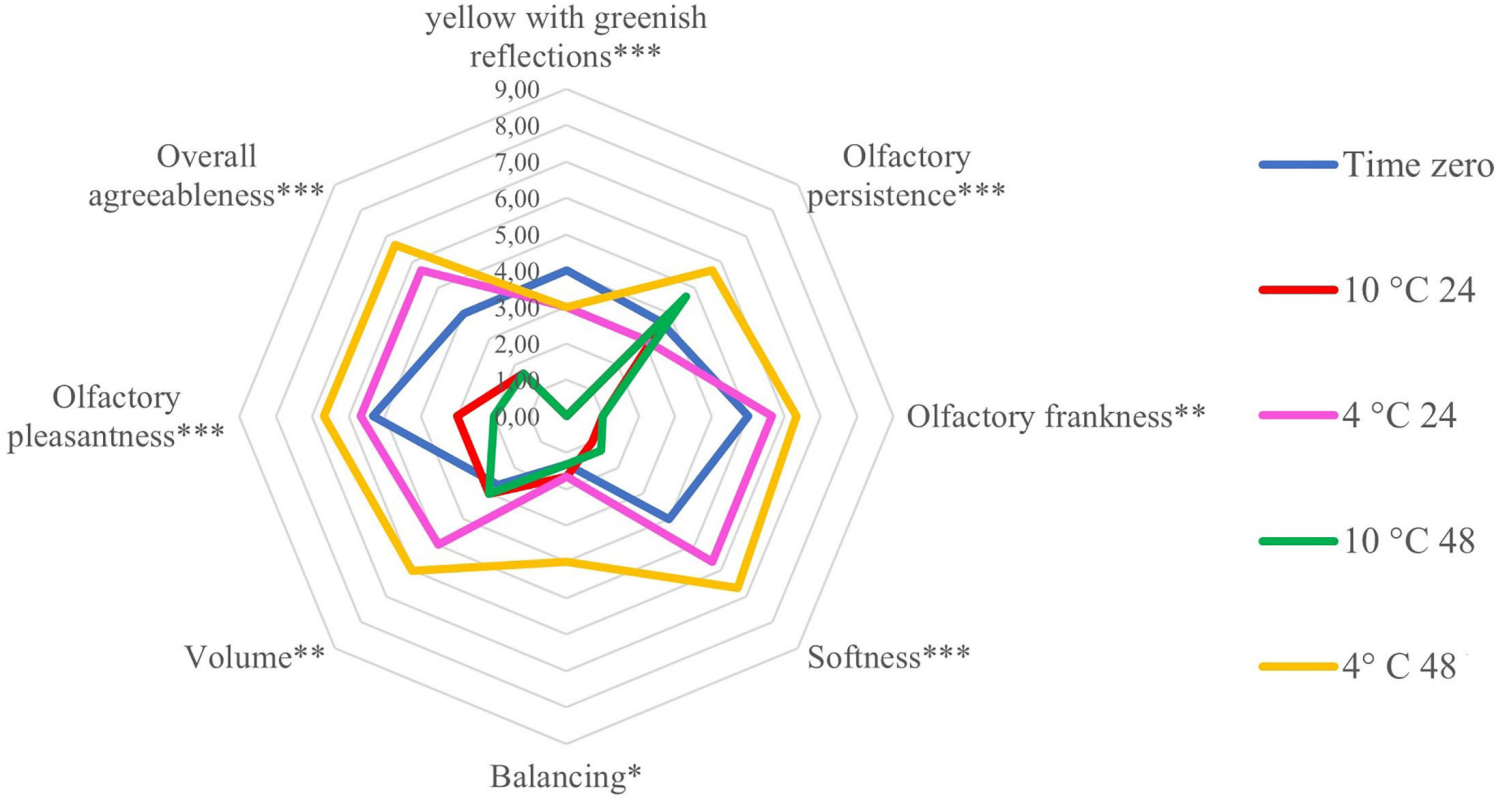
Sensorial parameters (yellow with greenish reflections, olfactory persistence, olfactory frankness, softness, balancing, volume, olfactory pleasantness, and overall agreeableness) of wines at the end of alcoholic fermentation. Wines were made from Vermentino grapes at the harvest (Time 0) and following cooling treatment at 4 and 10°C for 24 and 48 h. Asterisks represent statistically significant differences (***p < 0.001; **p < 0.01; *p < 0.05) based on ANOVA and Tukey *post-hoc* test performed between treatments and T0 wines.

Two different sensory profiles have been identified by the trained panel after analyzing the five wine samples (Supplementary Table 9; Figure 6). All average data for the evaluated attributes are reported in Supplementary Table 9, and Figure 6 shows only the statistically different parameters between the analyzed samples. The wine obtained using the T0 grapes was characterized by average frankness and olfactory pleasantness, low balance and volume, and greenish reflections values higher than those detected in 10°C samples. On the other hand, wines obtained with grapes refrigerated at 10°C showed, when compared with other samples, higher olfactory persistence and low values of softness, balance, volume, and overall agreeability. Wines obtained with the pre-treatment at 4°C on the contrary, were characterized by a high olfactory frankness, softness, balance, and volume higher than all the other wines. Finally, the hedonic descriptors, indicators of the general pleasantness, were higher in the wine produced with grapes cooled at 4°C for 24 h.

## Discussion

The composition of the grapes is one of the key factors to consider in order to obtain top quality wines. The general features of the bunches and of the single berries at harvest markedly affect all parameters applied to classify and describe wines, namely color, aroma, and overall flavor. The berry composition is the result of metabolic processes occurring throughout development, particularly during ripening, strongly modulated by internal (physiological, inter/intra-organ competition) and external (agronomic, environmental) factors. Considering the climatic parameters, it is well recognized that high temperatures occurring after veraison affect berry primary and secondary metabolism resulting in altered ripening process and, under some circumstances, unbalanced composition. This is a condition more frequently occurring in several grape growing areas due to the climate change, often resulting in summers characterized by extreme conditions. The exposition of berries to high temperature during ripening has marked effects on composition, including those on aromatic potential that appears to be reduced through the deregulation of specific genes. Tomás Matus et al. (3) demonstrated that Cabernet Sauvignon berries kept at high temperature (about 35°C) during the last stages of ripening show a repression of genes encoding for enzymes involved in terpenoids biosynthetic pathway, such as 1-deoxy-D-xylulose-5-phosphate synthase, terpene synthase, geraniol 10-hydroxylase, (–)-germacrene D synthase, and linalool synthase. High temperature from veraison to harvest, and, in particular, the bunch zone air temperature, resulted to be negatively correlated with the concentration of the sesquiterpene rotundone in cv Shiraz grape berry and wine (21). Despite scarce direct experimental data available, the effects of high temperatures on the decrease of aroma compounds of grapes due also (and extensively) to oxidative processes are widely accepted. In other fruit species, such as mandarin, the application of short-term or intermitted high temperature (38°C) immediately after harvest for pathogen control purposes (curing) results in oxidation of limonene and linalool (22). Thus, it can be expected that cooling fruit immediately after harvest is an effective method to counteract these oxidative losses. As for wine grapes, cooling before processing (pre-cooling) is a practice applied during grapes transportation from the field to the cellar or after the arrival of the grapes in the cellar. This is done to remove heat from the berries thus avoiding the loss of VOCs and heat-related oxidative processes with obvious advantages in terms of aroma traits of the resulting wines according to Mencarelli and Bellincontro (23). Our results indeed show that the pre-cooling of harvested Vermentino grapes for 24 or 48 h at 4 or 10°C is effective in altering the VOC profile of the berries, with, as a general effect, an increase of specific compounds belonging to the terpenoid category, and specifically to the sesquiterpene subclass. Terpenoids content in wine grapes is considered crucial for grapes and wine aroma. For instance, monoterpenes are the main responsible for the aromatic traits of “aromatic” and Muscat-like varieties (24). In the recent years, due to the increased temperatures, terpenoids content in grapes is strongly decreasing (4), and this has a marked impact on the quality of the resulting wines, lowering the overall wine quality. In fact, other studies report that with the increase of temperature, terpenoids are easily oxidized to less aromatic compounds, thus resulting in a general decrease of terpenoids content (25). Together with monoterpenes and isoprenoids, sesquiterpenes are the terpenoids of major importance in grape (24) and are recognized to be co-responsible for the organoleptic characteristics of wines (26). Compared to the other categories, less attention has been addressed to sesquiterpenes due to their lower volatility and higher odor thresholds (27). One of the best described sesquiterpene compound is rotundone, responsible for the “black pepper” organoleptic trait in Shiraz wine, formed by oxidation of the precursor α-guaiene (28). Even if there is only limited research on sesquiterpene production and impact in grapes, this category of terpenoid is considered to provide balsamic, spicy, and woody notes (29). In addition, based on recent evidence, sesquiterpenes have been proposed to induce health-beneficial effects, based on their anti-inflammatory and antimicrobial properties (28, 30). The observed effect of pre-cooling on sesquiterpene content in grapes (and wines) could be the result of a synergistic effect in terms of reduced oxidation and new production due to the induction of the terpenoid biosynthetic pathway. In fact, cubebene and cadinene, considering the 48 and 24 h treatments, respectively, showed significant increases in their content in grapes compared to both T0 and respective controls, and gurjunene resulted significantly higher in 10°C in 48 samples. In other products and plant species (toon buds, *Toona sinensis* Roem), limited (up to 3 days) cold storage (at 4°C) resulted in an increase of different volatile compounds including sesquiterpenes (31). These authors also state that the observed high expression levels of genes involved in the terpenoid backbone biosynthesis pathways contributed to sesquiterpene production after cold storage. Our molecular data indicate that *VvHDR* gene, representing a key step in the biosynthesis of precursors of mono and sesquiterpenes, shows an up-regulation in cooled samples compared to T0 grapes, and an increasing trend compared to control, suggesting an induction of the terpenoid pathway. The up-regulation of *VvTER* gene reinforces the hypothesis of an activation of a specific pathway related to the production of monoterpenes, an effect of great interest for aromatic and Muscat-like varieties. If a change in monoterpene metabolism has an impact on, or a relation with, sesquiterpenes, it still remains to be elucidated through improvements of our, so far, limited knowledge and understanding on sesquiterpene accumulation processes and biosynthetic mechanisms in grape berries.

Compared with grapes at harvest, cooled and control samples showed a general decrease of titratable acidity. In harvested grapes, the respiration process proceeds, and carbohydrate and organic acid consumption occurs, thus probably resulting in lower acidity values (32). The sugar content reached the highest value in control grapes, which is consistent with the higher weight loss rate reached by this grape considering that higher is the water loss higher is the concentration of soluble in berries. Cooled grapes resulted in higher polyphenols content, while controls reached the lowest values. In our previous and preliminary study carried out on the same variety with the same experimental setup (12), similar but not always consistent behavior was observed. Specifically, in both years, refrigerated grapes at 10°C for 24 h reached the highest value of TPC and control grapes after 48 h had the lowest. Polyphenols biosynthesis is exerted via phenylpropanoid pathway which is well known to be highly affected by both external and internal factors and involved in the response to different stresses in plants (33, 34), including acclimation to different temperatures (35). In grapes, this pathway is particularly important considering that polyphenols play a key role in grape and wine quality traits, such as color intensity and stability, body, and astringency. In our study, *VvPAL*, which is the key and first gene of the pathway, was up-regulated, suggesting an important effect of factors such as detachment and postharvest life on the specific expression. Additionally, the increased levels of total polyphenols at lower temperature, along with up-regulation of other genes, such as *VvSTS2* and *VvFLS1*, suggest that pre-cooling treatments also induce the activation of specific branches of the phenylpropanoids pathways. This finding is consistent with previous studies which observed an induction of the initial stage of the phenylpropanoid pathway and of specific branches responsible for stilbenes and flavonoids biosynthesis following exposure of table grape berries to cold stress under aerobic or short-term anaerobic conditions (36–38). As far as wine grapes is considered, Mencarelli et al. (39) reported an up-regulation of *PAL* and *STS* during controlled postharvest dehydration at 10°C, in correspondence of 10% water loss. Here, the up-regulation of these genes occurred even before the 10% of weight loss. Even though our findings on phenylpropanoid pathway-related genes expression and metabolites accumulation show good consistency, contradictory results were observed for *PPO* gene expression. *PPO* enzymes are responsible for polyphenols oxidation, and they are well known to be stimulated during different postharvest protocols, especially at a higher temperature. Mencarelli et al. (39) showed that, in harvested wine grapes, the temperature of 20°C increased *PPO* activity while temperatures below 10°C reduced its expression. In our case, the highest expression level of *PPO* was observed in grapes kept at 10°C, paralleled by increases in TPC. Suehiro et al. (40) reported that in grape berries, the up-regulation of *PPO* is often accompanied with the induction of genes involved in polyphenols biosynthesis (i.e., *STS, CHS*, and *FLS)* to rebalance polyphenols content.

The modification of the grapes composition has an impact on the quality of the resulting wine. The alcoholic fermentation trend did not show any substantial difference among samples. The only difference was observed in terms of duration: the fermentation of musts from cooled grapes finished earlier, probably due to the lower sugar content of the starting grapes. Consistently, wine made starting from cooled grapes had a lower alcohol degree. Additionally, cooling down the temperature of grapes, results in wines characterized by higher malic acid content and acidity values. Acidity parameters are, nowadays, extremely important for white wine quality. They play a key role in mouthfeel and flavor, and acts as a buffer to preserve the wine for longer. However, climate change and shifts in seasonal temperatures, warmer-than-normal, often lead to overripe grapes with low level of acids, especially malic acid, and high sugars concentration (41), thus, resulting in an overall unbalanced wine. Low malic acid content, especially in white wines that do not undergo malolactic fermentation, may result in tartaric acid addition (42). Therefore, the applied pre-processing cooling treatment could ensure adequate acidity values resulting in improvement of wine freshness.

Wine technological parameters and volatile profiles are affected by a high number of factors related to the composition of the grapes and the winemaking protocols. The higher values of titratable acidity and malic acid in the wines made from pre-cooled grapes are indeed of great interest, considering the effects of high temperatures on primary metabolism during the final stages of ripening, leading to rapid decreases of the organic acidity (malate) in the berries. Specifically, considering the wine aroma, grape-growing factors (cultivar, soil type, water availability and canopy management), winemaking techniques (pre-crushing treatments, crushing methods, must treatment and maceration), and the interaction between them strongly influence the final volatile profile (43, 44). Additionally, many volatiles are formed during fermentation due to yeast and malolactic bacteria metabolism, further modifying the wine aroma. Acetic acid was the only volatile compound significantly affected by the cooling treatments. Volatile acetic acid is mainly produced during fermentation by lactic acetic bacteria metabolism (45). Excessive quantities of acetic acid above the olfactory threshold are generally associated to acrid taste and unpleasant vinegar aroma (45, 46). In the present study, even though pre-cooling of grapes is associated with higher acetic acid, the related volatile acidity resulted lower, and the sensory profile of these wines did not show any acetic and unpleasant characters. Therefore, the increase of volatile acetic acid was probably still lower than the odor threshold value of this molecule despite statistical significance. The prevalence of specific classes of volatiles contributes the most to different and specific aroma traits in wine (47). For instance, terpenoid family, which, in our study, was the only class of volatiles increased in wines made starting from refrigerated grapes, has been correlated with fruity and floral aroma (26). Interestingly, different temperatures increased accumulation of different subclasses of terpenoids. Pre-cooling at 10°C significantly increased the content of sesquiterpenes, a class of terpenoids which is still not well characterized in terms of specific contribution to wine aroma and flavor (27). On the other hand, in grapes pre-cooled at 4°C, monoterpenes show an increasing trend of accumulation. This subclass of terpenoid is probably the most studied due to its impact on specific flavor and aroma wine traits (27), such as floral, fruity, and citrus flavors (48). Previous studies also demonstrated an increase of terpenoids in wine made from refrigerated grapes of the white-skinned variety (11). The Vermentino wines made with pre-cooled grapes at 4°C showed a general better sensory profile (i.e., high olfactory frankness, mouthfeel, balance and volume) reaching after 48 h the highest rate of overall agreeableness. This finding is in agreement with results presented by Mafata et al. (10), who report that the lowest pre-cooling temperature of grapes (<10°C) led to wine described as balanced and well developed. In addition to the changes of the VOC profiles, the different sensory profiles described for the 4°C wine samples should be considered also in relation to the lower pH and higher acidity, parameters markedly affecting the sensorial properties of the wines.

## Conclusion

Our analytical data and sensory evaluations show that the temperature of grapes entering the vinification process can strongly influence the aroma and the overall quality traits of the resulting wines. The detected effects of the applied pre-cooling treatments in cv Vermentino appear to be associated with specific changes occurring in terms of VOC profiles, particularly considering the class of terpenoids, in general, and the sub-class of sesquiterpenes. Further investigations are needed to better define and characterize the impact of sesquiterpenes on the overall wine aroma and flavor in wines produced with different grapes (aromatic, neutral), styles, and vinification techniques. If the changes detected in VOC profile following pre-cooling are the result of changes in the production and/or the degradation remains to be elucidated. The higher acidity levels detected in wines produced with pre-cooled grapes should be considered of interest for winemakers operating in areas characterized by high temperatures at harvest.

## Data Availability Statement

The raw data supporting the conclusions of this article will be made available by the authors, without undue reservation.

## Author Contributions

PT conceived the experiments and coordinated the trials. RS, MMo, MMa, AB, and SB performed the grapes and wine analyses. FV coordinated the sensory panel. PT, MMo, SB, FV, and AZ analyzed the data and discussed the results. MMo and PT wrote the first draft of the manuscript. SB, FV, and AZ wrote specific sections of the manuscript. All authors contributed to manuscript revision, read, and approved the submitted version.

## Conflict of Interest

The authors declare that the research was conducted in the absence of any commercial or financial relationships that could be construed as a potential conflict of interest.

## Publisher's Note

All claims expressed in this article are solely those of the authors and do not necessarily represent those of their affiliated organizations, or those of the publisher, the editors and the reviewers. Any product that may be evaluated in this article, or claim that may be made by its manufacturer, is not guaranteed or endorsed by the publisher.

## References

[1] Jones GV White MA Cooper OR Storchmann K. Climate change and global wine quality. Clim Change. (2005) 73:319–43. 10.1007/s10584-005-4704-2

[2] Rienth M Torregrosa L Sarah G Ardisson M Brillouet JM Romieu C. Temperature desynchronizes sugar and organic acid metabolism in ripening grapevine fruits and remodels their transcriptome. BMC Plant Biol. (2016) 20:16. 10.1186/s12870-016-0850-0 27439426 PMC4955140

[3] Tomás Matus J Vannozzi A Pastenes C Lecourieux D Kappel C Lecourieux F. Dissecting the biochemical and transcriptomic effects of a locally applied heat treatment on developing cabernet sauvignon grape berries. Front. Plant Sci. (2017) 1:53. 10.3389/fpls.2017.00053 28197155 PMC5281624

[4] Ribéreau-Gayon P Dubourdieu D Doneche B Lonvaud A. (2006). Handbook of enology. Volume 1. The Microbiology of Wine and Vinifications. 2nd ed. West Sussex: John Wiley and Sons Ltd.

[5] Tonutti P. Innovations in storage technology and postharvest science. Acta Hortic. (2013) 1012, 323–30. 10.17660/ActaHortic.2013.1012.4029792573

[6] Heredia FJ Escudero-Gilete ML Hernanz D Gordillo B Meléndez-Martínez AJ Vicario IM. Influence of the refrigeration technique on the colour and phenolic composition of syrah red wines obtained by pre-fermentative cold maceration. Food Chem. (2010) 118:377–83. 10.1016/j.foodchem.2009.04.13Hikaru

[7] Marais J. Effect of grape temperature, oxidation and skin contact on Sauvignon blanc juice and wine composition and wine quality. South African J Enol Vitic. (2017) 19:10–6. 10.21548/19-1-2238

[8] Marais J. Effect of grape temperature and yeast strain on Sauvignon blanc Wine Aroma composition and quality. South African J Enol Vitic. (2001) 22:47–50. 10.21548/22-1-2168

[9] Marais J. Effect of different wine-making techniques on the composition and quality of pinotage wine. I. Low-temperature skin contact prior to fermentation. South African J Enol Vitic. (2003) 24:70–5. 10.21548/24-2-2642

[10] Mafata M Buica A du Toit W Panzeri V van Jaarsveld FP. The effect of grape temperature on the sensory perception of méthode cap classique wines. South African J Enol Vitic. (2018) 39:132–140. 10.21548/39-1-2620

[11] Coletta C Botondi R Forniti R Baccelloni S Bellincontro A Mencarelli F. Alternating temperature in postharvest cooling treatment of Fiano and Falanghina grapes affects cell wall enzyme rate, berry softening and polyphenols. J Sci Food Agric. (2019) 99:3142–8. 10.1002/jsfa.9529 30537182

[12] Modesti M Shmulevitz R Brizzolara S Tonutti P. Short term low temperature treatments of harvested wine grapes (cv Vermentino) affect the volatile organic compound profile of the berries. Adv Hort Sci. (2020) 34:27–33. 10.13128/ahsc-7855

[13] Singleton VL Rossi JA. Colorimetry of total phenolics with phosphomolybdic-phosphotungstic acid reagents. Am J Enol Vitic. (1965) 16:144–158.

[14] Venturi F Andrich G Quartacci MF Sanmartin C Andrich L Zinnai A. A kinetic method to identify the optimum temperature for β-glucanase activity. South African J Enol Viticul. (2013) 34:281–6. 10.21548/34-2-1106

[15] Sanmartin C Taglieri I Venturi F Ferroni G Flamini G Macaluso M. (2019). Co-fermentation of intact grape clusters and stalk: a natural and economical strategy to modulate nutraceutical and sensory features of Syrah variety. Agrochimica. (2019) 63:197–207. 10.12871/00021857201927

[16] OIV. Compendium of International Analysis of Methods. (2006). OIV (Resolution Oeno 1/2006)

[17] Tonacci A Billeci L di Mambro I Marangoni R Sanmartin C Venturi F. Wearable sensors for assessing the role of olfactory training on the autonomic response to olfactory stimulation. Sensors. (2021) 21:770. 10.3390/s21030770 33498830 PMC7865293

[18] Livak KJ Schmittgen TD. Analysis of relative gene expression data using real-time quantitative PCR and the 2–ΔΔCT method. Methods. (2001) 25:402–8. 10.1006/meth.2001.1262 11846609

[19] Genova G Montanaro G. Qualitative Evaluation of Aroma-Active Compounds in Grape and Grape-Derived Products by Means of Headspace SPME-GC/MS Analysis. (2012). Branford, CT: Perkin Elmer.

[20] OIV. 18/73 and Eco 3/2003: Musts and wines-OIV definition (2003).

[21] Zhang P Howell K Krstic M Herderich M Barlow E Fuentes S. Environmental factors and seasonality affect the concentration of rotundone in *Vitis vinifera* L. cv. Shiraz Wine. PLoS ONE. (2015) 10:133137. 10.1371/journal.pone.0133137 26176692 PMC4503395

[22] Pérez AG Luaces P Oliva J Ríos JJ Sanz C. Changes in vitamin C and flavour components of mandarin juice due to curing of fruits. Food Chem. (2005) 91:19–24. 10.1016/j.foodchem.2004.05.041

[23] Mencarelli F Bellincontro A. Recent advances in postharvest technology of the wine grape to improve the wine aroma. J Sci Food Agric. (2020) 100:5046–55. 10.1002/jsfa.8910 29369355

[24] Lin J Massonnet M Cantu D. The genetic basis of grape and wine aroma. Hortic Res. (2019). 6:81. 10.1038/s41438-019-0163-1 31645942 PMC6804543

[25] Ribereau-Gayon P Boidron JN Terrier A. Aroma of Muscat grape varieties. Agric Food Chem. (1975) 23:1042–7. 10.1021/jf60202a050

[26] D'Onofrio C Matarese F Cuzzola A. Study of the terpene profile at harvest and during berry development of *Vitis vinifera* L. aromatic varieties Aleatico, Brachetto, Malvasia di *Candia aromatica* and *Moscato bianco*. J Sci Food Agric. (2017) 97:2898–907. 10.1002/jsfa.8126 27801497

[27] Black CA Parker M Siebert TE Capone DL Francis IL. Terpenoids and their role in wine flavour: recent advances. Aust J Grape Wine Res. (2015) 21:582–600. 10.1111/ajgw.12186

[28] Li Z Howell K Fang Z Zhang P. Sesquiterpenes in grapes and wines: occurrence, biosynthesis, functionality, and influence of winemaking processes. Compr Rev Food Sci Food Saf. (2020) 19:247–81. 10.1111/1541-4337.12516 33319521

[29] Della cassa E Neo YP Iammarino M Ugliano M Slaghenaufi D. Norisoprenoids, sesquiterpenes and terpenoids content of valpolicella wines during aging: investigating aroma potential in relationship to evolution of tobacco and balsamic aroma in aged wine. Frontiers in Chem. (2018) 6:66. 10.3389/fchem.2018.00066 29616214 PMC5867301

[30] Perestrelo R Silva C Pereira J Câmara JS. Healthy effects of bioactive metabolites from Vitis vinifera l. Grapes: a review. In Grapes: production, phenolics composition and potential biomedical effects. (2015). New York, NY: Nova Science Technology, 335–8.

[31] Zhao H Feng S Zhou W Kai G. Transcriptomic analysis of postharvest toon buds and key enzymes involved in terpenoid biosynthesis during cold storage. Sci Hort. (2019) 257:108747. 10.1016/j.scienta.2019.108747

[32] Rehman MA Khan MR Sharif MK Ahmad S. Study on the storage stability of fruit juice concentrates. Pak J Food Sci. (2014) 24:101–107.

[33] Tuccio L Remorini D Pinelli P Fierini E Tonutti P Scalabrelli G. Rapid and non-destructive method to assess in the vineyard grape berry anthocyanins under different seasonal and water conditions. Aust J Grape Wine Res. (2011) 17, 181–9 10.1111/j.1755-0238.2011.00139.x

[34] Dixon A Lahoucine A Parvathi K Chang-Jun L Srinivasa R Liangjiang W. The phenylpropanoid pathway and plant defence-a genomics perspective. Plant Cell. (2002) 6:1085–1097. 10.1105/tpc.7.7.1085 20569344

[35] Solecka D Kacperska A. Phenylpropanoid deficiency affects the course of plant acclimation to cold. Physiol Plant. (2003) 119:253–262. 10.1034/j.1399-3054.2003.00181.x

[36] Maoz I de Rosso M Kaplunov T Dalla Vedova A Sela N Flamini R. Metabolomic and transcriptomic changes underlying cold and anaerobic stresses after storage of table grapes. Sci Rep. (2019) 9:2917. 10.1038/s41598-019-39253-8 30814549 PMC6393478

[37] Moral J Kolukisaoglu U Amâncio S Teresa Sanchez-Ballesta M Rosales R Romero I. Low temperature and short-term high-CO_2_ treatment in postharvest storage of table grapes at two maturity stages: effects on transcriptome profiling. Front Plant Sci. (2016) 7:1020. 10.3389/fpls.2016.01020 27468290 PMC4942463

[38] Sanchez-Ballesta MT Romero I Jiménez JB Orea JM González-Ureña Á Escribano MI. Involvement of the phenylpropanoid pathway in the response of table grapes to low temperature and high CO_2_ levels. Postharvest Biol Technol. (2007) 46:29–35. 10.1016/j.postharvbio.2007.04

[39] Mencarelli F Bellincontro A Nicoletti I Cirilli M Muleo R Corradini D. Chemical and biochemical change of healthy phenolic fractions in winegrape by means of postharvest dehydration. J Agric Food Chem. (2010) 58:7557. 10.1021/jf100331z 20521817

[40] Suehiro Y Mochida K Itamura H Esumi T. Skin browning and expression of PPO, STS, and CHS genes in the grape berries of ‘Shine Muscat.' *J Japn Soc Hortic Sci*. (2013) 83:122–32. 10.2503/jjshs1.ch-095

[41] Santisi J. Warming Up the Wine Industry. Environ Mag. (2011) 22:15–17.27748529

[42] Keller M. Managing grapevines to optimise fruit development in a challenging environment: a climate change primer for viticulturists Aust J Grape Wine Res. (2009) 16:56–69. 10.1111/j.1755-0238.2009.00077.x

[43] Ribereau-Gayon P Glories Y Maujean A Dubourdieu D. The Chemistry of Wine Stabilization and Treatments. In: Handbook of Enology. 2nd edn (2000). Chichester: John Wiley and Sons, p. 497

[44] Henschke P Costello PJ Bartowsky E. Management of malolactic fermentation: wine flavour manipulation. Aust NZ Grapegrow Winemak. (2002) 461:7–14.

[45] Swiegers J Bartowsky E Henschke P Pretorius I. Yeast and bacterial modulation of wine aroma and flavour. Aust J Grape Wine Res. (2005) 11:139–73. 10.1111/j.1755-0238.2005.tb00285.x

[46] Mas A Torija MJ del Carmen García-Parrilla M Troncoso AM Chen YM Suh SJ. Acetic acid bacteria and the production and quality of wine vinegar. Sci World J. (2014) 2014:394671. 10.1155/2014/394671 24574887 PMC3918346

[47] De-La-Fuente-Blanco A Sáenz-Navajas MP Ferreira V. On the effects of higher alcohols on red wine aroma. Food Chem. (2016) 210:107–14. 10.1016/j.foodchem.2016.04.021 27211627

[48] Mateo JJ Jiménez M. Monoterpenes in grape juice and wines. J Chromatogr. (2000) 881:557 567. 10.1016/s0021-9673(99)01342-4 10905735

