# Supplementary material for: Pre-processing Cooling of Harvested Grapes Induces Changes in Berry Composition and Metabolism, and Affects Quality and Aroma Traits of the Resulting Wine
Source: Front Nutr. 2021 Nov 24;8:728510. doi: 10.3389/fnut.2021.728510 (PMC8652203; doi:10.3389/fnut.2021.728510)
Supplement: Supplementary file 1 [file Data_Sheet_1.pdf]

Supplementary Material

Table S1. First order descriptors utilized to define the not structured parametric descriptive scoring chart and their definitions.

**View attributes**	**Definitions**
Yellow with greenish reflections	Presence of perceptible amounts of yellow pale color with greenish reflections typical for young white wines.
Turbidity	Degree of turbidity (presence of haze-causing colloids or particulate matter); can vary from clear to dull to cloudy.
**Smell attributes**	
Fineness of smell	Finely balanced character of whole smell attributes of the wine.
Odor intensity	Quantity of odorants compounds as perceived by the assessor.
Olfactory frankness	Absence of any off flavors. in smell of wine.
Olfactory persistence	The olfactory perceptions that linger in the headspace of the glass after filling.
**Taste attributes**	
Acidity	A sour perception derived as a complex response to organic acids. wine pH. and the sensory impact of other sapid substance. notably sugars. ethanol. and phenolic compounds; flat refers to the absence of sufficient acidity. the opposite of acidic; tart usually denotes an appropriate. pleasant acidic perception.
Softness	Complex response to compounds such as sugars. glycerol. and ethanol. as influenced by sensations to the acidic and phenolic compounds in the wine; cloying refers to an intense. unpleasant sensation of sweetness; the opposite is dry.
Bitter	A perception induced primarily by the presence of small molecular weight phenolic compounds that is influenced marginally by the presence of sugars. ethanol. and acids.
Body	The summary perception of weight or richness in the mouth; a tactile sensation induced primarily by the presence of alcohol. but clearly influenced by the presence of sugars. glycerol (in high concentration). and phenolics; full-bodied is a positive perception of weight in the mouth; watery is the negative perception of the absence of sufficient body.

Volume	Tactile sensation induced primarily by the presence of alcohol. but clearly influenced by the presence of sugars. glycerol (in high concentration). and phenolics; full-bodied is a positive perception of weight in the mouth; watery is the negative perception of the absence of sufficient body.
Balancing	The perception of harmony. notably between the sweet. sour. bitter. and astringent sensation in the mouth. but clearly influenced by the intensity of the aromatic sensation of the wine; one of the most highly regarded of wine attributes.
**Hedonic parameters**	
Visual pleasantness	Recognition and enjoyment of the good visual qualities of the wine.
Olfactory pleasantness	Recognition and enjoyment of the good olfactory qualities.
Taste pleasantness	Recognition and enjoyment of the good taste qualities.
Overall agreeableness	Recognition and enjoyment of the good qualities of the wine evaluated on the whole.

Table S2. Gene name. forward and reverse sequence. GenBank accession number. reaction temperature and primer efficiency (%) of the genes studied in this work.

Gene name	Forward primer sequence	Reverse primer sequence	GenBank	Reaction temperature	Efficiency (%)
VvPAL	CTGGCCAAATCGAGGCTGC	CTTCTGCATCAGTGGATATGTG	XM_003633937.3	62	97
VvFLS1	AAACCACCTACTTACAGAG	ACCTAACCCCAGTGACAGAC	XM_002285803.4	60	93
VvTER	GGGAATGCTCTGCTTGTAC	TTCCAAGATGTCTGTGTGG	NM_001281134.1	60	95
VvPPO	CCCATATTCTTGGTCACCAC	GCGTCAAGCCAATCCGTATC	NM_001281116.1	62	93
VvGT14	CCTCAAGAGCAAGTTCTCA	CACTGTCTATCTCCATTCCTAC	XM_002285734.2	67	110
VvDXS3	TTGAAAGGGAAACGGGAAC	TGGGTGTAAAGAATGACGACT	XM_002282392.3	57	90
VvHDR	CGTTATGTTAGTAGTTGGTG	CTTATTCTGTTTCCTGGACCTA	XM_002284623.3	62	91
VvSTS2	GGGAAGGAAGCAGCATTGA	GGCATTTCTACACCCGAGG	XM_003634020.3	62	90
VvACT7	GCATCCCTCAGCACCTTCCA	CCACCTCAACACATCTCCATGT	XM_002282480.4	67	109

Table S3. One-way ANOVA and Tuckey post-hoc test performed on VOCs detected in grapes (*Vitis vinifera L.* cv Vermentino) at harvest (Time 0) and after 24 h at 4. 10 and 22 (control) °C.

Compound	f. value	p. value	Tuckey post-hoc test
Cadinene	22.453	5.6003E-6	4 °C **a**; 10 °C **b**; Control **c**; Time 0 **c**
Hexadecanoic acid	22.248	5.9378E-6	4 °C **a**; 10 °C **b**; Control **c**; Time 0 **c**
Butadiene	22.019	6.3413E-6	4 °C **b**; 10 °C **b**; Control **a**; Time 0 **a**
2-Heptanone	17.146	2.977E-5	4 °C **a**; 10 °C **a;** Control **a**; Time 0 **b**
1-Hexanol	9.0871	9.572E-4	4 °C **b**; 10 °C **b**; Control **a**; Time 0 **a**
Hexanal	6.7225	0.0038123	4 °C **b**; 10 °C **a**; Control **b**; Time 0 **a**
Cubebene	4.6486	0.016048	4 °C **a**; 10 °C **ab**; Control **ab**; Time 0 **b**
2-Hexenal	3.9884	0.026831	4 °C **ab**; 10 °C **a**; Control **ab**; Time 0 **b**
2-methyl-2-Propanol	3.9559	0.02754	4 °C **ab**; 10 °C **b**; Control **ab**; Time 0 **a**
Benzoic acid	3.9012	0.028781	4 °C **ab**; 10 °C **ab**; Control **a**; Time 0 **b**

Table S4. One-way ANOVA and Tuckey post-hoc test performed on VOCs detected in grapes (*Vitis vinifera L.* cv Vermentino) at harvest (Time 0) and after 48 h at 4. 10 and 22 (control) °C.

Compound	f. value	p. value	Tuckey post-hoc test
2-Heptanone	41.168	9.4728E-8	4 °C **a**; 10 °C **a**; Control **a**; Time 0 **b**
Hexadecanoic acid	27.231	1.5974E-6	4 °C **ab**; 10 °C **a**; Control **a**; Time 0 **b**
Gurjunene	18.323	1.9922E-5	4 °C **b**; 10 °C **a**; Control **b**; Time 0 **b**
Cubebene	17.112	3.0123E-5	4 °C **a**; 10 °C **a**; Control **b**; Time 0 **b**
2-Hexenal	5.3491	0.0096023	4 °C **ab**; 10 °C **a**; Control **a**; Time 0 **b**

Table S5. pH. titratable acidity (g/L tartaric acid). malic acid (g/L). alcohol degree (% V/V). volatile acidity (g/L) and polyphenols content (mg/L GAE) of the musts at the beginning of the fermentation process.

T=0	Sugar content (g/l hexose)	Ph	Titratable acidity (g/l tartaric acid)	Malic acid (g/l)	Polyphenols content (mg/l GAE)
T0	220.34 ± 2.12 a	3.46 ± 0.02 a	5.01 ± 0.03 b	1.75 ± 0.06 c	586 ± 43 c
10 °C 24	211.55 ± 1.13 b	3.43 ± 0.02 b	4.91 ± 0.05 c	1.92 ± 0.05 b	804 ± 49 a
10 °C 48	194.23 ± 1.43 d	3.42 ± 0.03 b	4.94 ± 0.05 bc	1.91 ± 0.04 b	846 ± 29 a
4 °C 24	195.52 ± 2.65 d	3.40 ± 0.03 b	5.76 ± 0.04 a	2.08 ± 0.03 a	673 ± 51 b
4 °C 48	201.16 ± 2.16 c	3.44 ± 0.02 ab	4.76 ± 0.04 d	2.14 ± 0.04 a	685 ± 36 b

Table S6. CIELAB parameters (L*. a*. b*. C*. h*) of the musts at the beginning of fermentation process.

t=0	L*	a*	b*	C*	h*
T0	85.57 ± 0.12 a	-0.86 ± 0.06 a	3.28 ± 0.03 b	3.39 ± 0.04 b	104.7 ± 0.1 a
10 °C 24	85.32 ± 0.13 a	-0.76 ± 0.07 b	3.45 ± 0.05 a	3.53 ± 0.06 a	102.4 ± 0.2 b
10 °C 48	86.45 ± 0.11 a	-0.74 ± 0.03 b	3.48 ± 0.06 a	3.55 ± 0.04 a	102.0 ± 0.2 c
4 °C 24	85.67 ± 0.15 a	-0.69 ± 0.03 b	3.35 ± 0.04 b	3.42 ± 0.03 b	101.6 ± 0.3 c
4 °C 48	85.60 ± 0.16 a	-0.70 ± 0.02 b	3.31 ± 0.04 b	3.38 ± 0.03 b	101.9 ± 0.1 c

Table S7 One-way ANOVA and Tuckey post-hoc test performed on VOCs identified in wines made from grapes (*Vitis vinifera L.* cv Vermentino) at harvest (Time 0) and after 24 h at 4 and 10 °C.

Compound	f. value	p. value	Tuckey post-hoc test
Acetic acid	17.572	2.7197E-4	4 °C **a**; 10 °C **b**; Time 0 **b**

Table S8 One-way ANOVA and Tuckey post-hoc test performed on VOCs identified in wines made from grapes (*Vitis vinifera L.* cv Vermentino) at harvest (Time 0) and after 48 h at 4 and 10 °C.

Compound	f. value	p. value	Tuckey post-hoc test
Acetic acid	68.977	2.6263E-7	4 °C **a**; 10 °C **a**; Time 0 **b**
Guaiene	14.663	5.9945E-4	4 °C **b**; 10 °C **a**; Time 0 **b**
Germacrene	13.439	8.6458E-4	4 °C **b**; 10 °C **a**; Time 0 **b**
Gurjunene	12.473	0.001174	4 °C **b**; 10 °C **a**; Time 0 **b**
Cubebene	8.6144	0.0047886	4 °C **b**; 10 °C **a**; Time 0 **b**
Cadinene	8.6144	0.0047887	4 °C **b**; 10 °C **a**; Time 0 **b**

Table S9. The average data of the different sensory descriptors for all the wine samples analyzed.

First order descriptor	Time zero	10°C 24	4°C 24	10°C 48	4°C 48
**Yellow with greenish reflections**	4.0±0.4 a	1.0±0.6 c	3.0±0.3 b	1.2±0.5 c	3.0±0.5 b
**Turbidity**	0.6±0.8 a	2.0±1.2 a	1.3±0.6 a	1.7±0.5 a	1.3±0.5 a
**Odor intensity**	6.7±1.0 a	6.0±0.5 a	7.0±0.8 a	5.7±1.5 a	7.7±0.9 a
**Fineness of smell**	4.3±0.5 a	3.3±0.6 a	4.0±0.5 a	3.3±0.5 a	5.3±0.7 a
**Olfactory persistence**	3.7±0.4 bc	4.0±0.5 b	3.0±0.3 c	4.7±0.6 ab	5.7±0.5 a
**Olfactory frankness**	5.0±0.5 a	1.0±0.4 b	5.7±0.3 a	1.0±0.5 b	6.3±0.5 a
**Acidity**	3.7±0.5 a	4.0±0.5 a	3.7±0.4 a	4.3±0.8 a	4.7±0.9 a
**Softness**	4.0±0.5 b	1.0±0.3 c	5.7±0.8 a	1.3±0.5 c	6.7±0.4 a
**Bitter**	4.3±0.5 a	4.7±0.8 a	4.3±0.6 a	4.0±0.7 a	5.3±0.7 a
**Balancing**	1.3±0.5 b	1.7±0.4 b	1.7±0.4 b	1.3±0.6 b	4.0±0.5 a
**Body**	3.3 ±0.3 a	3.0±0.5 a	3.7±0.5 a	3.3±0.7 a	4.5±1.0 a
**Volume**	2.7±0.5 b	3.0±0.3 b	5.0±0.6 a	3.0±0.5 b	6.0±0.5 a
**Visual pleasantness**	4.7±0.5 a	4.0±0.8 a	4.3±0.6 a	3.0±1.3 a	5.3±1.1 a
**Olfactory pleasantness**	5.3±0.4 b	3.0±0.6 c	5.7±0.5 ab	2.0±0.5 c	6.7±0.5 a
**Taste pleasantness**	3.3±1.2 a	2.7±0.5 a	4.0±0.9 a	2.7±0.8 a	4.3±1.5 a
**Overall agreeableness**	4.0±0.8 b	1.7±0.5 c	5.7±1.0 ab	1.7±0.4 c	6.7±0.8 a
